# Validation of markers with non-additive effects on milk yield and fertility in Holstein and Jersey cows

**DOI:** 10.1186/s12863-015-0241-9

**Published:** 2015-07-22

**Authors:** Hassan Aliloo, Jennie E. Pryce, Oscar González-Recio, Benjamin G. Cocks, Ben J. Hayes

**Affiliations:** Biosciences Research Division, Department of Economic Development, Jobs, Transport and Resources, AgriBio, 5 Ring Road, Bundoora, VIC 3083 Australia; School of Applied Systems Biology, La Trobe University, Bundoora, VIC 3083 Australia; Dairy Futures Cooperative Research Centre (CRC), AgriBio, 5 Ring Road, Bundoora, VIC 3083 Australia

**Keywords:** Non-additive genetic effect, Fertility, Dairy cow, Genome-wide association study

## Abstract

**Background:**

It has been suggested that traits with low heritability, such as fertility, may have proportionately more genetic variation arising from non-additive effects than traits with higher heritability, such as milk yield. Here, we performed a large genome scan with 408,255 single nucleotide polymorphism (SNP) markers to identify chromosomal regions associated with additive, dominance and epistatic (pairwise additive × additive) variability in milk yield and a measure of fertility, calving interval, using records from a population of 7,055 Holstein cows. The results were subsequently validated in an independent set of 3,795 Jerseys.

**Results:**

We identified genomic regions with validated additive effects on milk yield on *Bos taurus* autosomes (BTA) 5, 14 and 20, whereas SNPs with suggestive additive effects on fertility were observed on BTA 5, 9, 11, 18, 22, 27, 29 and the X chromosome. We also confirmed genome regions with suggestive dominance effects for milk yield (BTA 2, 3, 5, 26 and 27) and for fertility (BTA 1, 2, 3, 7, 23, 25 and 28). A number of significant epistatic effects for milk yield on BTA 14 were found across breeds. However on close inspection, these were likely to be associated with the mutation in the *diacylglycerol O-acyltransferase 1* (*DGAT1*) gene, given that the associations were no longer significant when the additive effect of the *DGAT1* mutation was included in the epistatic model.

**Conclusions:**

In general, we observed a low statistical power (high false discovery rates and small number of significant SNPs) for non-additive genetic effects compared with additive effects for both traits which could be an artefact of higher dependence on linkage disequilibrium between markers and causative mutations or smaller size of non-additive effects relative to additive effects. The results of our study suggest that individual non-additive effects make a small contribution to the genetic variation of milk yield and fertility. Although we found no individual mutation with large dominance effect for both traits under investigation, a contribution to genetic variance is still possible from a large number of small dominance effects, so methods that simultaneously incorporate genotypes across all loci are suggested to test the variance explained by dominance gene actions.

**Electronic supplementary material:**

The online version of this article (doi:10.1186/s12863-015-0241-9) contains supplementary material, which is available to authorized users.

## Background

Female fertility is of great interest to the dairy industry because impaired reproductive ability can reduce the profitability of a dairy herd, particularly by increased expenses of additional inseminations, veterinary treatments and replacement cows [1, 2]. Selection to improve milk production traits in Holstein and Jersey cattle populations has led to a decline in fertility traits in the last few decades due to unfavourable genetic correlations between fertility and milk production [3]. Many countries have now included fertility in their national breeding goals [4, 5], however fertility related traits usually have low heritability estimates [3, 6, 7], and genetic improvement through traditional breeding programs is slow, although substantial genetic variation exists [8].

When heritability estimates are low for a trait, one could examine the non-additive part of genetic variation for opportunities to improve the trait of interest. Non-additive genetic variation is the result of allele by allele interactions and involves intra-locus (dominance) and inter-locus (epistasis) interactions. Pedigree based estimates of non-additive genetic variance for fertility related traits have been reported to be as large as or larger than additive variance [9]. Hoeschele [10] estimated additive and non-additive genetic variance for a number of cow fertility measures in US Holsteins and obtained broad sense heritabilities that were at least twice as large as narrow sense heritabilities, albeit with large standard errors. Similarly, Fuerst and Solkner [9] reported a higher proportion of the phenotypic variance explained by dominance and additive × additive epistatic effects than heritability estimated in the narrow sense for calving interval (CI). Druet *et al.* [11] observed similar values for additive and dominance variances in analyses of fertility traits for Austrian Simmental and Brown Swiss dairy cattle and Palucci *et al.* [12] estimated non-additive genetic effects of sizable magnitude for a number of fertility measures in Canadian Holstein heifers and cows and suggested including non-additive genetic effects in models for estimating genetic merit of animals.

The prediction of non-additive genetic effects is not a trivial task and requires complex statistical and computational methods [13]. In traditional genetic evaluation methods, pedigrees are usually not informative enough to accurately estimate non-additive genetic effects for each individual and in many cases these effects are confounded with other non-genetic effects such as common environment, or maternal effects that may lead to over-estimation [12, 14]. The use of genomic data instead of pedigree information has the potential to overcome these problems when both phenotypes and genotypes for individuals in a given population are known. Availability of genotypes coupled with phenotypes has led to a renewed interest in the estimation of non-additive genetic variance. Sun *et al.* [15] showed that dominance variance can account for up to 7 % of total phenotypic variance of yield traits in dairy cattle and including additive and dominance effects in the model fits data better than including only additive effects. Ertl *et al.* [16] obtained larger estimates of dominance variance for milk production and conformation traits in Fleckvieh cattle such that the ratio of dominance variance over total genetic variance ranged from 3.3 % to 50.5 % in their study.

Over the last decade, high throughput genotyping has provided a valuable source of information to study the relationships between phenotypes and genotypes in livestock breeding [17, 18]. Access to large SNP arrays at an affordable price has made genome-wide association studies (GWAS) a common practice. GWAS use linkage disequilibrium (LD) between DNA markers and QTL to identify variants associated with traits and it can be used to map QTL regions throughout the genome [19]. Genome-wide association studies can be used to estimate both the additive and non-additive effects of genetic markers, but most published GWAS for dairy cattle to date have focused on additive effects of genes while non-additive interactions are generally neglected, with a few exceptions (*e.g.* [15]). This might not be an appropriate assumption since the modes of biological actions are often more complicated than can be explained by simple additive models [20]. Known genotypes of individuals are more informative than pedigree based methods, especially for estimating dominance effects. The disadvantage comes from the large increase in dimensionality generated by including all potential epistatic interactions. Testing combinations of all possible allelic interactions would be ideal, however it is not always computationally feasible and the results might not be interpretable. An alternative approach to decrease the dimensionality is to perform a filtering step in which a set of variants or genes are selected and subsequently tested for epistatic effects [21–23].

The objective of this study was to detect chromosomal regions with additive and non-additive genetic effects for calving interval and milk yield (MY) using a Holstein discovery population. We then attempted to validate these associations in an independent Jersey population of cows. The benefits and limitation of accounting for non-additive effects in genetic analyses are discussed, with examples from the present study.

## Results

### Additive marker effects

**Fig. 1:**
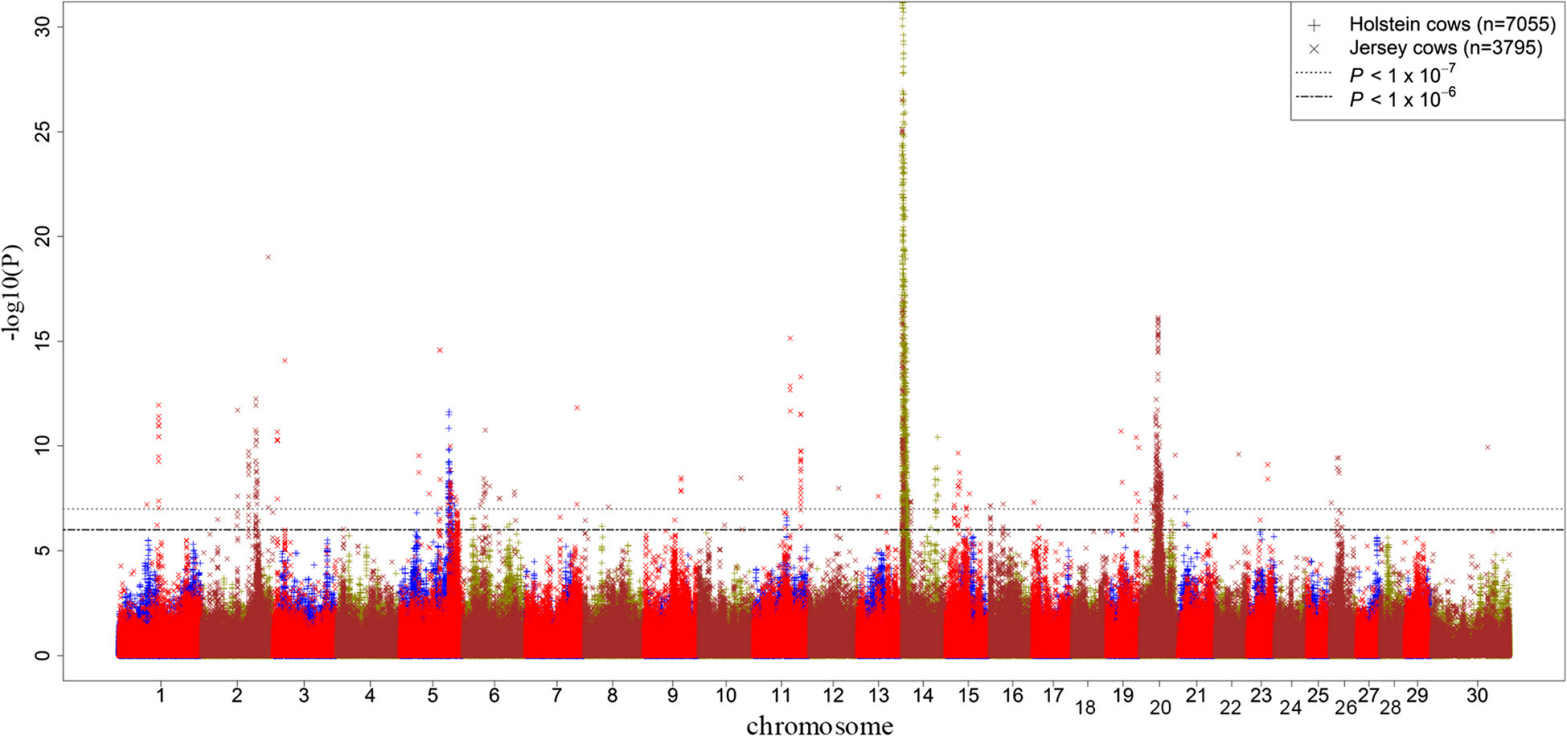
Distribution of additive SNP effects for milk yield. Manhattan plot of all additive SNP effects for milk yield in discovery and validation populations with chromosome number on horizontal axis and –log_10_(*P-*value) on vertical axis

**Fig. 2:**
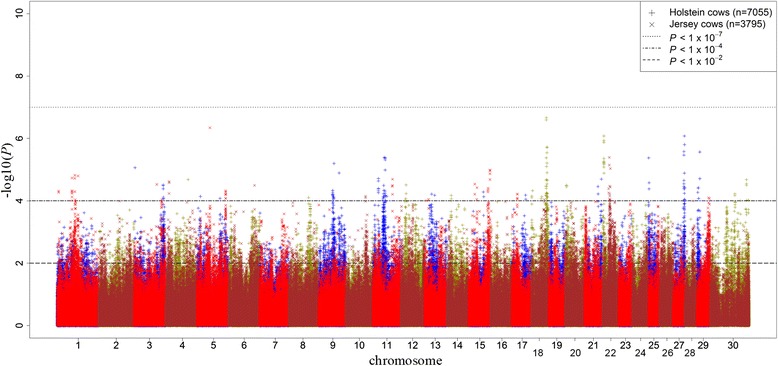
Distribution of additive SNP effects for fertility. Manhattan plot of all additive SNP effects for calving interval in discovery and validation populations with chromosome number on horizontal axis and –log_10_(*P-*value) on vertical axis

Manhattan plots of all additive SNP effects for MY and CI in study populations are presented in Figs 1 and 2. There were a large number of SNPs for MY that reached the 5 % genome-wide significance level after the Bonferroni correction in both Holstein (*P* < 1 × 10^−7^) and Jersey (*P* < 1 × 10^−5^) populations. However, for CI (Fig. 2) very few SNPs passed this threshold, therefore lower suggestive thresholds in Holstein (*P* < 0.0001) and Jersey (*P* < 0.01) cows were set to identify potential associations. Significant associations for MY were found on BTA 5, 14 and 20 whereas suggestive additive SNP effects associated to CI were observed on BTA 5, 9, 11, 18, 22, 27, 29 and X chromosome.

In the association analysis of MY, 715 SNPs were significant (*P* < 1 × 10^−7^) in the Holstein analysis (Table 1). The number of SNPs which validated in the Jersey population at the probability threshold of *P* < 1 × 10^−5^ was 93 in the individual SNP validation but this increased to 413 in the segment validation (Table 1). Out of the 93 individually validated additive SNP effects on MY, 64 had the same direction in both discovery and validation populations. False discovery rates (FDR) for MY were calculated to be very close to zero in all cases. For CI, 136 SNPs were significant (*P* < 0.0001) in the Holstein set which corresponds to an estimated FDR of 30 %, while only 5 of these were found to be significant (*P* < 0.01) in the Jersey population used for validation, with an estimated FDR equal to 26 % (Table 1). All of the 5 validated effects were in the same direction in Holstein and Jersey cows. In segment SNP validation for CI, the number of significant SNPs (*P* < 0.01) was 73 with a FDR of 1 %.

**Table 1:** P-value thresholds, number of SNPs found to be additively significant and corresponding false discovery rates (FDR) for milk yield (MY) and calving interval (CI) in discovery and validation populations

	Discovery			Individual validation				Segment validation		
Trait	*P*-value threshold	Holstein discovery (7055)	FDR (%)	*P*-value threshold	Jersey validation (3795)	FDR (%)	No. Same Dir.^a^	*P*-value threshold	Jersey validation (3795)	FDR (%)
MY	10^−7^	715	0	10^−5^	93	0	64	10^−5^	413	0
CI	0.0001	136	30	0.01	5	26	5	0.01	73	1

^a^Number of same direction SNP effects in discovery and validation populations

### Dominance marker effects

**Fig. 3:**
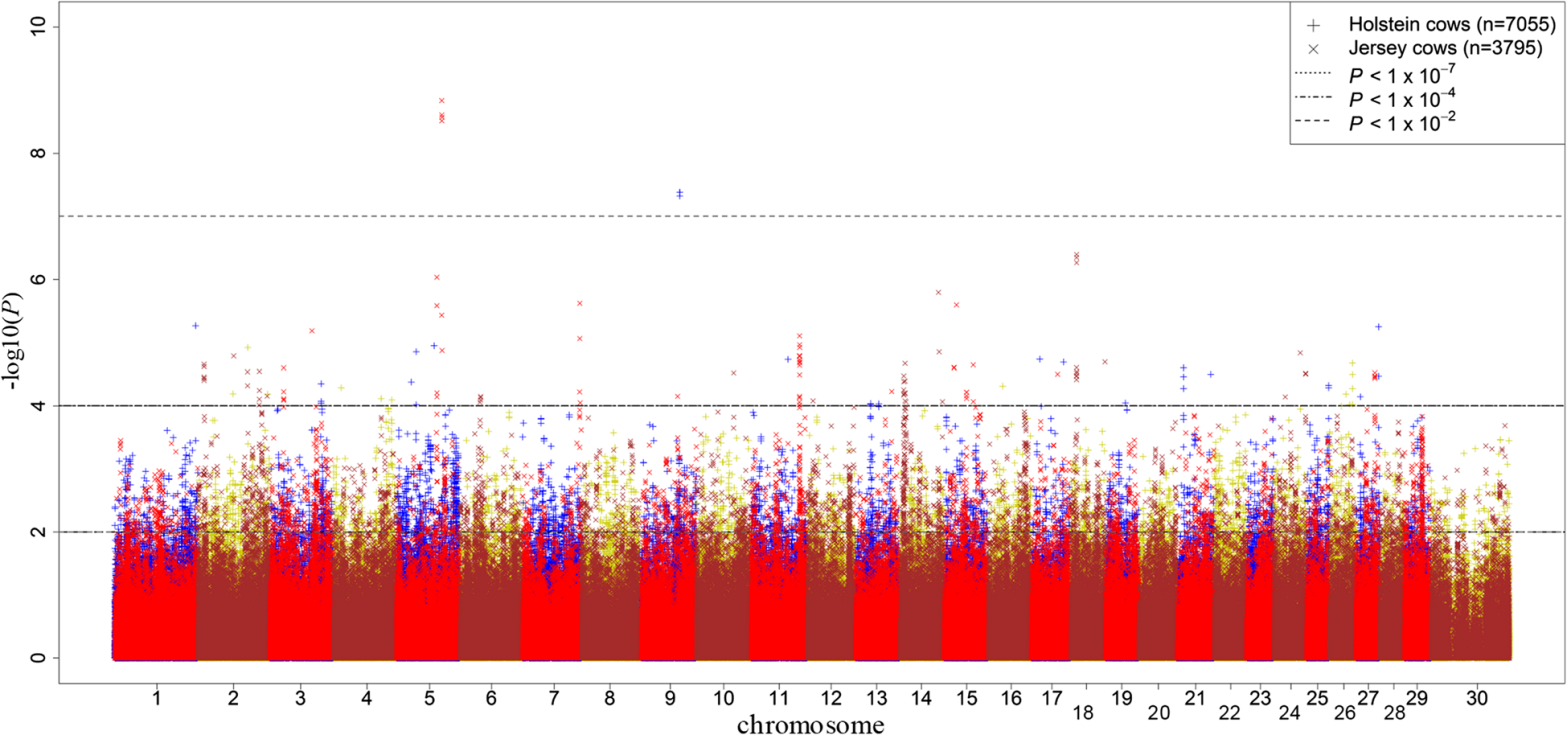
Distribution of dominance SNP effects for milk yield. Manhattan plot of all dominance SNP effects for milk yield in discovery and validation populations with chromosome number on horizontal axis and –log_10_(*P-*value) on vertical axis

**Fig. 4:**
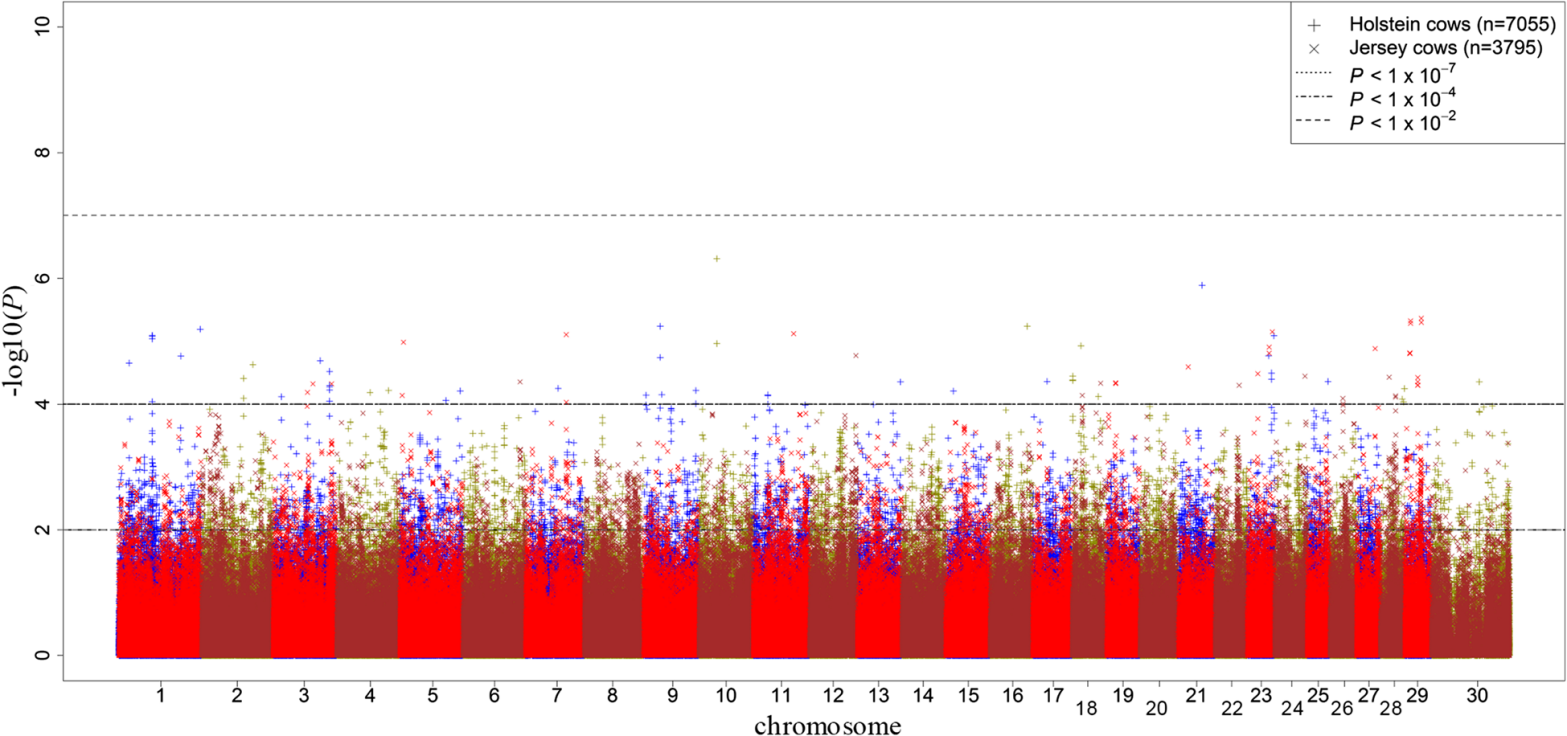
Distribution of dominance SNP effects for fertility. Manhattan plot of all dominance SNP effects for calving interval in discovery and validation populations with chromosome number on horizontal axis and –log_10_(*P-*value) on vertical axis

The Manhattan plots of all dominance SNP effects for MY and CI are shown in Figs. 3 and 4 respectively. No dominance effects were found to be significant for either MY or CI at the genome-wide threshold of *P* < 1 × 10^−7^ (5 % Bonferroni corrected). Therefore for both traits, SNP effects were tested with a suggestive less stringent threshold of *P* < 0.0001 in Holstein population and validated in Jersey cows at a threshold of *P* < 0.01.

The magnitude of significant dominance effects were smaller than those for additive effects especially for MY. This was confirmed with large FDRs, such that in the discovery analyses of both traits, these values were calculated to be more than 100 % (Table 2). Forty SNPs were significant (*P* < 0.0001) in the Holstein discovery population for MY, but only 1 of these was validated (*P* < 0.01) in Jersey cows with different signs observed in the discovery and validation analyses and with a FDR of 39 % (Table 2). The number of validated SNPs increased to 21 using the segment validation approach (*P* < 0.01) with a FDR equal to 1 %. For CI, 36 SNPs were found to have significant (*P* < 0.0001) dominance associations in the Holstein discovery set (Table 2). Of these, 3 (1 with same direction) and 10 SNPs were validated respectively in individual (FDR = 11 %) and segment (FDR = 3 %) validations in the Jersey population when a threshold of *P* < 0.01 was applied. The validated SNPs with suggestive significant dominance effects on MY and CI were detected on 5 (BTA 2, 3, 5, 26 and 27) and 7 (BTA 1, 2, 3, 7, 23, 25 and 28) chromosomes, respectively.

**Table 2:** P-value thresholds, number of SNPs with significant dominance effects and corresponding false discovery rates (FDR) for milk yield (MY) and calving interval (CI) in discovery and validation populations

	Discovery			Individual validation				Segment validation		
Trait	*P*-value threshold	Holstein discovery (7055)	FDR (%)	*P*-value threshold	Jersey validation (3795)	FDR (%)	No. Same Dir.^a^	*P*-value threshold	Jersey validation (3795)	FDR (%)
MY	0.0001	40	102	0.01	1	39	0	0.01	21	1
CI	0.0001	36	113	0.01	3	11	1	0.01	10	3

^a^Number of same direction SNP effects in discovery and validation populations

### Epistasis interactions

There were 255,255 and 9,180 pairwise interaction effects included in the epistasis analyses for MY and CI respectively. The SNPs were selected where they had significant additive effects in the discovery population of Holsteins (Table 1). We only performed individual validation for epistatic analyses, such that if an interaction between two SNPs was significant in discovery population we checked for its significance also in validation set.

Similar to the additive analysis, a larger number of pairwise interactions were found to be statistically significant for milk yield compared with fertility (Table 3). However, since all of the SNPs that had validated interactions for MY were located at the beginning of BTA 14 and near *diacylglycerol O-acyltransferase 1* (*DGAT1*) gene, we suspected that these interactions may have been due to the *DGAT1* mutation effect [24]. Therefore, the epistatic model was extended to include the *DGAT1* effect to see if any of the interactions remained significant. We did this by including an additional SNP effect in the model, this SNP was the highest peak in the *DGAT1* region. The absence of significant interactions in this region after including the SNP in high LD with the *DGAT1* effect in the model suggests that the identified significant pairwise interactions were picking up the *DGAT1* effect by creating haplotype like combinations. That is, the linkage disequilibrium of SNP allele combinations with the *DGAT1* mutation was higher than for the individual SNP.

**Table 3:** P-value thresholds, number of significant pairwise additive × additive interactions and calculated false discovery rates (FDR) for milk yield (MY) and calving interval (CI) in discovery and validation populations

		Discovery			Validation			
Trait	No. of tested pairwise interactions	*P*-value threshold	Holstein discovery (7055)	FDR (%)	*P*-value threshold	Jersey validation (3795)	FDR (%)	No. Same Dir.^a^
MY	255,255	10^−7^	3700	0	10^−5^	165	0	163
CI	9,180	10^−4^	5	18	0.01	0	NA	NA

^a^Number of same direction SNP effects in discovery and validation populations

Five additive × additive interactions were found that had significant (*P* < 0.0001) effects on CI in Holstein analysis with a FDR of 18 %, however none of them was validated (*P* < 0.01) in the Jersey cows, so we did not report them here.

## Discussion

Additive and non-additive genetic variants influencing MY and CI were identified using a large GWAS applied to phenotypes and genotypes of females of two breeds of dairy cattle. We found several significant additive and dominance associations in a discovery population of Holstein cows which were confirmed in Jersey cows. Although many of the additive QTL effects identified and validated here overlapped with previously reported genomic regions in the literature, this study is novel in identifying a number of QTL regions associated with dominance effects on MY and CI.

We discovered more additive associations for MY than for CI, which was likely a consequence of the higher heritability of MY. Calving interval is the most widely used measure of female fertility in national genetic evaluations worldwide [3, 8], but it has a low heritability. Besides, CI suffers from censored records where data from cows unsuccessful to calve again are excluded from evaluations, or a maximum arbitrarily value is assigned which impairs the predictive ability of models not accounting for censoring [25]. These contribute to the low power in detecting underlying additive genetic variants. Other detailed measures of fertility as well as potential biomarkers linked with female reproduction [8, 26] which have higher heritabilities could provide a better insight of the genetic underlying female fertility in future.

High false discovery rates in identifying SNPs with dominance effects on both MY and CI in the discovery population of Holsteins (Table 2) indicates that the identified associations might only serve as a reference for future studies. Increasing the number of observations would improve power.

### Locating QTL regions

#### Additive effects

Four regions were detected that had additive associations with MY, of which 2 were on BTA 5 and 1 each on BTA 14 and BTA 20 (Table 4). The longest (~4 Mbp) region was on chromosome 14, extended from 1.4 to 5.3 Mbp and comprised 96 % of the significant additive SNP associations. All of the individually validated SNPs for MY, except 1 on BTA 5, were also found within this region, where 65 of them associated with 31 genes (Additional file 1: Table S1). A cluster of genes with suggested effects on all milk production traits in dairy breeds have previously been identified in this region (*e.g.* [27–29]). The most significant association in this interval in both Holstein and Jersey animals was SNP rs109421300 (~1.8 Mbp) located within an intron of the *DGAT1* gene. This marker also had the largest additive effect on MY and explained highest proportion of phenotypic variance (5.694 %) by additive effects for MY. The effect of *DGAT1* on several production traits including MY has been previously demonstrated in several studies [24, 30, 31].

**Table 4:** Boundaries of the validated regions with significant additive effects on milk yield and the most significant SNPs within the identified regions with their associated genes in discovery and validation populations

		Most strongly associated SNP in discovery						Most strongly associated SNP in validation						
BTC^a^	Interval (Mbp)^b^	SNP	Position (bp)	-log10 (P)	Effect ± SE	MAF^c^	$\frac{\sigma_a^2}{\sigma_p^2}$ (%)^d^	SNP	Position (bp)	Effect ± SE	-log10 (P)	MAF	$\frac{\sigma_a^2}{\sigma_p^2}$ (%)	Genes^e^
5	94.453 - 95.026	rs136374794	94518850	11.633	120.3 ± 17.11	0.300	0.490	rs136816685	95001236	−80.76 ± 16.46	6.013	0.471	0.459	PTPRO
5	**96.927 - 97.854**	rs110729080	97435197	8.791	149.8 ± 24.79	0.117	0.373	rs134869818	97031962	97.04 ± 16.57	8.291	0.452	0.660	GPRC5A^f^
14	**1.428 - 5.289**	rs109421300	1801116	134.354	−389.4 ± 15.34	0.369	5.694	rs109421300	1801116	−234.1 ± 17.53	38.947	0.468	3.858	**DGAT1** ^**f**^
20	29.568 - 30.367	rs134175348	30001269	7.230	−104.6 ± 19.27	0.206	0.289	rs42276093	29568029	−133.9 ± 19.25	11.382	0.270	0.999	NA

^a^BTC: *Bos Taurus* chromosome

^b^Intervals containing individually validated SNPs are in bold

^c^ *MAF* minor allele frequency

^d^ *σ*_*a*_^2^ = additive variance; *σ*_*p*_^2^ = phenotypic variance

^e^Genes with both top SNPs in discovery and validation inside them are in bold

^f^Genes with individually validated SNPs within them

Chromosome 5 contained 2 significant regions for MY (94.5 to 95.0 Mbp and 96.9 to 97.9 Mbp) which extended beyond the previously reported significant regions on this chromosome for milk production traits [32, 33]. Wang *et al.* [32] reported a significant region between 91.2 Mbp and 97.1 Mbp for milk fat percentage, with the most significant SNP located in an intron of the *epidermal growth factor receptor pathway substrate 8* (*EPS8*) gene. This gene has also been reported by Raven *et al.* [33] as influencing milk yield in Australian Holstein and Jersey populations. In our study, the two aforementioned regions contained the most significant SNPs rs136816685 at 95.0 Mbp (Jersey) and rs110729080 at 97.4 Mbp (Holstein) respectively inside intronic regions of *protein tyrosine phosphatase, receptor type, O* (*PTPRO*) gene and *G protein-coupled receptor, family C, group 5, member A* (*GPRC5A*) gene. *PTPRO* is located 18 Kbp downstream of *EPS8* so it is likely that the same QTL affecting milk production traits is responsible for detected associations in these studies. A candidate gene in the other interval, *GPRC5A*, is associated with signal transduction between cells and has been reported as having differential expression (up regulation / turning on) during the onset of lactation in bovine mammary tissue [34].

The region on BTA 20 for MY was located on the middle of this chromosome which was strongly suggested as having a QTL affecting milk production traits [35, 36]. A mutation in the *Growth hormone receptor* (*GHR*) gene has been suggested as underlying the QTL in this region [35].

Seventeen significant regions suggesting several genes with additive effects on CI were discovered in this study (Table 5). Chromosome 18 had the highest number of significant regions for CI but BTA 9 and 27 contained more significant associations than other chromosomes with each having about 22 % of significant SNPs. The most significant additive effect for fertility was SNP rs41996522 (−log10(*P*) = 6.076) located on BTA 22 which also explained the highest proportion (0.137 %) of phenotypic variance for CI by additive effects. Nevertheless, all of the individually validated SNPs were found within the region on X chromosome extending from 139.2 to 139.8 Mbp.

**Table 5:** Boundaries of the validated regions that are additively significant on calving interval as well as the most significant SNPs and their associated genes within these regions in discovery and validation populations

		Most strongly associated SNP in discovery						Most strongly associated SNP in validation							
BTC^a^	Interval (Mbp)^b^	SNP	Position (bP)	-log10 (P)	Effect ± SE	MAF^c^	$\frac{\sigma_a^2}{\sigma_p^2}$ (%)^d^	SNP		Position (bp)	-log10 (P)	Effect ± SE	MAF	$\frac{\sigma_a^2}{\sigma_p^2}$ (%)	Genes
5	12.551 - 13.463	rs133249083	13027942	4.139	2.462 ± 0.620	0.329	0.095	rs135584613		13270757	3.229	−3.077 ± 0.893	0.325	0.140	NA
5	88.607 - 89.159	rs135833682	88822777	4.076	−2.191 ± 0.556	0.417	0.083	rs133539520		88861488	2.679	−2.784 ± 0.934	0.313	0.112	ABCC9
9	55.233 - 55.657	rs134339497	55233033	4.325	−2.364 ± 0.580	0.402	0.096	rs136630637		55637401	2.399	−2.559 ± 0.887	0.337	0.098	NA
9	57.397 - 57.735	rs137407787	57396816	4.130	−2.346 ± 0.591	0.358	0.091	rs42550144		57723628	2.408	−2.685 ± 0.929	0.290	0.100	EPHA7
9	60.121 - 60.477	rs43600502	60477358	5.195	−2.642 ± 0.584	0.352	0.114	rs133175600		60130790	2.127	2.813 ± 1.05	0.212	0.087	LOC101902479
11	20.620 – 21.274	rs133774241	20994163	4.318	−2.706 ± 0.665	0.241	0.096	rs137059194		20898669	2.410	−2.467 ± 0.8529	0.458	0.101	LOC783737
11	39.466 - 39.772	rs109315341	39466071	4.126	−2.971 ± 0.749	0.177	0.092	rs133126268		39747182	2.408	−3.706 ± 1.284	0.115	0.094	NA
11	40.896 - 41.299	rs133462686	41298588	4.491	−3.036 ± 0.729	0.182	0.098	rs109834745		40895791	2.414	3.554 ± 1.228	0.139	0.101	LOC101903002
18	4.541 - 4.810	rs109920290	4541123	4.326	2.4 ± 0.589	0.391	0.098	rs110689012		4810082	2.044	3.37 ± 1.29	0.119	0.080	NA
18	37.446 - 37.925	rs41875426	37446338	4.061	2.679 ± 0.682	0.213	0.086	rs137407722		37925382	3.142	4.111 ± 1.213	0.147	0.142	NA
18	53.789 - 54.605	rs41891477	54232476	4.180	−2.302 ± 0.576	0.399	0.091	rs109907036		54028686	3.680	4.065 ± 1.094	0.186	0.168	PRKD2 / PPP5C
18	57.109 - 58.052	rs110801791	57516245	4.675	−3.508 ± 0.824	0.136	0.103	rs41895542		57269152	2.496	3.6 ± 1.219	0.133	0.100	NA
18	61.922 - 62.150	rs133761590	62115202	4.661	2.525 ± 0.594	0.369	0.106	rs137170802		62143810	2.736	−3.018 ± 0.966	0.255	0.116	CACNG6 / VSTM1
22	4.979 - 5.598	rs41996522	5028345	6.076	−2.784 ± 0.564	0.442	0.137	rs41995585		5133660	2.168	−3.028 ± 1.117	0.168	0.086	NA
27	41.873 - 42.109	rs134294374	42079983	5.581	−2.76 ± 0.586	0.367	0.127	rs41586304		41872925	2.295	3.105 ± 1.107	0.176	0.094	NR1D2
27	43.595 - 44.261	rs110746407	43914360	6.075	−2.781 ± 0.564	0.441	0.136		rs43064076	43595406	2.873	−3.831 ± 1.192	0.146	0.123	NA
X	**139.211 - 139.509**	rs136627433	139508886	4.679	−2.606 ± 0.6098	0.394	0.116		rs110719178	139490243	2.320	−3.327 ± 1.175	0.163	0.101	NA

^a^BTC: *Bos Taurus* chromosome

^b^Intervals containing individually validated SNPs are in bold

^c^ *MAF* minor allele frequency

^d^ *σ*_*a*_^2^ = additive variance; *σ*_*p*_^2^ = phenotypic variance

Most of the identified QTL regions for CI in this study have been previously reported in the literature. Chromosome 18 has been largely investigated in search for QTLs affecting reproduction traits in dairy breeds [37–39]. Sahana *et al.* [39] found strongest marker associations for some direct calving traits on this chromosome on a region ranging from 55.2 to 60.0 Mbp in Danish and Swedish Holstein cattle. Their detected interval covers one of the identified QTL regions in the present study (57.1 – 58.1 Mbps). The most strongly associated SNP in this region found by these authors, which was previously reported by Cole *et al.* [17] as having largest effect on several traits including calving ease, was located in an intron of the *sialic acid binding Ig-like lectin-5* (*SIGLEC5*) gene. *SIGLEC5* was suggested to have a role on the initiation of parturition in Human [40], hence suggested influencing fertility in cattle [17]. Although we have not identified the same gene here, but *SIGLEC5* is positioned in our reported QTL region, so it may be possible that the described intervals are harbouring the same QTL.

Hoglund *et al.* [41] performed a large GWAS in a population of Nordic Holsteins for eight female fertility traits and validated their results in independent populations of Nordic Reds and Jerseys. They found several significant SNP associations for number of inseminations per conception in heifers and cows (BTA 5 and 11), Nordic female fertility index and length of the interval from calving to first insemination (BTA 9) and 56-day non-return rate in cows and heifers (BTA 27) which all overlapped with QTL regions for CI identified in our study.

Studies for detecting associations on chromosome X for fertility related traits are scarce in the literature and this chromosome is generally discarded in GWA studies mainly due to the use of mixed-sex observations. All of the SNPs that were individually validated in Jerseys for CI were found on the X chromosome, so future studies including data on this chromosome are suggested to search for QTL affecting fertility traits.

### Dominance effects

Of the 7 validated regions with dominance effects on MY, 3 were on BTA 26 and 1 identified on each of BTA 2, 3, 5 and 27 (Table 6). Chromosome 3 (97.9 – 98.8 Mbp) contained more significant SNPs (59 %) than other chromosomes but the identified region on BTA 5 (71.8 Mbp) encompassed the only individually validated SNP (rs110106971) with dominance effects on MY which happened to be within an intronic region of the *synapsin III* (*SYN3*) gene. The *ATP/GTP binding protein like-4* (*AGBL4*) gene was associated with both of the most significant SNPs in Holstein discovery (rs43361287) and Jersey validation (rs43363311) populations on the identified region on BTA 3 (97.9 – 98.8 Mbp). *AGBL4* was reported as a gene under positive selection in the dual purpose (milk and beef) Normande breed cattle [42]. Among the candidate genes on the 3 identified regions on BTA 26, *phospholysine phosphohistidine inorganic pyrophosphate phosphatase* (*LHPP*) gene was reported as a differentially expressed gene in mammary gland of Holstein-Friesian dairy cows affected by the polymorphism in *DGAT1* [43].

**Table 6:** Boundaries of the validated regions with significant dominance effect on milk yield as well as the most significant SNPs and their associated genes within these regions in discovery and validation populations^a^

		Most strongly associated SNP in discovery						Most strongly associated SNP in validation						
BTC^a^	Interval (Mbp)^b^	SNP	Position (bp)	-log10 (P)	Effect ± SE	MAF^c^	$\frac{\sigma_d^2}{\sigma_p^2}$ (%)^d^	SNP	Position (bp)	-log10 (P)	Effect ± SE	MAF	$\frac{\sigma_d^2}{\sigma_p^2}$ (%)	Genes^e^
2	95.312 - 95.730	rs136022579	95312328	4.920	155 ± 35.39	0.166	0.149	rs134324850	95725812	3.563	132 ± 36.23	0.171	0.197	ADAM23
3	97.907 - 98.799	rs43361287	98306933	4.351	82.29 ± 20.14	0.443	0.133	rs43363311	97907057	2.516	64.04 ± 21.6	0.427	0.139	**AGBL4**
5	**71.878 - 71.878**	rs110106971	71878168	4.947	−99.82 ± 22.71	0.332	0.158	rs110106971	71878168	2.012	55.58 ± 21.49	0.438	0.106	**SYN3** ^**f**^
26	32.249 - 32.341	rs42460360	32248251	4.185	129.5 ± 32.42	0.186	0.124	rs42741343	32336734	2.065	61.42 ± 23.36	0.349	0.110	LOC100847832
26	39.358 - 39.765	rs132810457	39358269	4.017	86.38 ± 22.13	0.343	0.122	rs110552548	39764774	2.239	−88.38 ± 32	0.214	0.125	GRK5
26	44.215 - 44.543	rs109406756	44537471	4.673	114.1 ± 26.83	0.244	0.143	rs134524557	44257893	2.246	70.3 ± 25.4	0.301	0.123	LHPP
27	42.674 - 42.890	rs41665573	42837186	5.248	−90.48 ± 19.92	0.469	0.164	rs41575082	42673983	2.328	62.09 ± 21.95	0.424	0.130	NA

^a^BTC: *Bos Taurus* chromosome

^b^Intervals containing individually validated SNPs are in bold

^c^MAF: minor allele frequency

^d^ *σ*_*d*_^2^ = dominance variance; *σ*_*p*_^2^ = phenotypic variance

^e^Genes with both top SNPs in discovery and validation inside them are in bold

^f^Genes with individually validated SNPs within them

There were 8 regions identified for CI (Table 7) suggesting some genes influencing fertility by dominance gene actions. The region on BTA 2 extended from 80.2 to 80.7 Mbp contained both of the most significant SNPs in Holsteins (rs41591067) and in Jerseys (rs133868000) within intronic regions of *Myosin IB* (*MYO1B*) gene. *MYO1B* associated with one of the individually validated SNPs (rs133868000) with dominance effect on CI in our study and was reported to have differential expression in *in vitro* culture of mouse blastocysts in suboptimal conditions [44]. Both of the top SNPs in Holstein (rs134910746) and Jersey (rs29020504) populations on the interval extended from 15.8 to 16.0 Mbp on BTA 3 were found to be inside intronic regions of *potassium intermediate/small conductance calcium-activated channel, subfamily N, member 3* (*KCNN3*) gene. *KCCN3* plays a key role in fluid secretion within the bovine oviduct which is essential to provide an appropriate environment for gamete maturation, transport, fertilization and early embryo development [45]. It has also been shown that *KCNN3* is differentially expressed between oocytes and granulosa cells (GCs) during development of the sheep ovarian follicle [46]. Similarly, human orthologue of *KCNN3* was reported as a gene associated with preterm birth [47].

**Table 7:** Boundaries of the validated regions with significant dominance effect on calving interval and the most significant SNPs with their associated genes within these regions in discovery and validation populations

		Most strongly associated SNP in discovery						Most strongly associated SNP in validation						
BTC^a^	Interval (Mbp)^b^	SNP	Position (bp)	-log10 (P)	Effect ± SE	MAF^c^	$\frac{\sigma_d^2}{\sigma_p^2}$ (%)^d^	SNP	Position (bp)	-log10 (P)	Effect ± SE	MAF	$\frac{\sigma_d^2}{\sigma_p^2}$ (%)	Genes^e^
1	**19.667 - 19.777**	rs110080440	19706636	4.651	−3.802 ± 0.896	0.299	0.091	rs109600947	19776964	2.178	−4.129 ± 1.52	0.249	0.080	NA
2	**80.202 - 80.654**	rs41591067	80201648	4.412	3.07 ± 0.746	0.500	0.084	rs133868000	80276795	2.403	−3.268 ± 1.133	0.473	0.089	**MYO1B** ^**f**^
3	15.808 - 15.963	rs134910746	15947344	4.119	3.024 ± 0.764	0.420	0.078	rs29020504	15808470	2.505	−5.434 ± 1.837	0.197	0.099	**KCNN3**
7	62.509 - 62.852	rs29013244	62508803	4.252	3.012 ± 0.747	0.480	0.081	rs43520270	62851917	2.123	3.664 ± 1.37	0.304	0.081	ABLIM3
23	46.082 - 46.581	rs137262994	46579868	4.494	−3.234 ± 0.777	0.407	0.087	rs109881533	46081778	2.401	4.222 ± 1.464	0.264	0.090	OFCC1
23	50.929 - 51.326	rs110165999	51326222	5.087	−5.153 ± 1.154	0.205	0.101	rs134147379	51081072	2.212	−3.689 ± 1.345	0.309	0.083	GMDS
25	39.070 - 39.921	rs135893130	39548382	4.364	−3.225 ± 0.788	0.390	0.084	rs108968775	39070284	2.808	3.583 ± 1.131	0.489	0.108	LOC618542
28	43.832 - 44.145	rs133899460	44144815	4.032	−2.918 ± 0.7461	0.480	0.076	rs109392728	43831664	2.404	−6.638 ± 2.302	0.143	0.089	CHAT

^a^ *BTC Bos Taurus* chromosome

^b^Intervals containing individually validated SNPs are in bold

^c^ *MAF* minor allele frequency

^d^ *σ*_*d*_^2^ = dominance variance; *σ*_*p*_^2^ = phenotypic variance

^e^Genes with both top SNPs in discovery and validation inside them are in bold

^f^Genes with individually validated SNPs within them

### Implications

One of the critical parameters determining the power of GWAS is the amount of LD between the observed SNP and the unobserved causal variant. In fact, the success of a GWAS in identifying QTLs with additive effects is controlled by *r*^*2*^ (r is the correlation between genetic marker and causative mutation) while detection of dominance or pairwise additive by additive effects depends on *r*^*4*^. This indicates there is a much higher reliance on LD when searching for non-additive effects compared to additive effects, if LD between the markers and QTL is incomplete [48]. This was reflected in results of the present study in which a larger number of markers with additive effects were identified than the markers with dominance and epistasis effects for both traits under investigation.

Although we validated some of the pairwise (putative epistatic) interactions for MY across breeds, a subsequent analysis that included the effect of the *DGAT1* gene, which has known effect on the trait [24], removed all of the detected associations. This suggests that the identified epistatic associations are actually haplotype effects that are in higher LD with the *DGAT1* mutation than the individual SNPs. This illustrates a problem with testing for epistatic interactions with common SNPs in imperfect LD with causative mutations; SNP by SNP interactions can describe haplotypes that are in higher LD with the causative mutation than the individual SNP, and are therefore significant when there is no true epistatic effect present. Putative epistatic interactions between common SNP should therefore be treated with caution.

The standard in reporting GWAS results is validation and before genotype-phenotype relationships can be used in selection decisions, they should be replicated in an independent population to confirm generalized effects in multiple populations [49]. Validation of GWAS results across breeds can refine QTL regions to narrower intervals [33] and is powerful in identifying positional candidate genes. This is because the extent of LD across cattle breeds is limited in contrast to within a breed where considerable LD can be maintained in intervals up to 1 Mbp as a result of a relatively small effective population size [50]. We validated a lower number of non-additive genetic associations than additive effects such that very few dominance effects for MY and CI were confirmed and no epistasis effect was common across Holstein and Jersey cows for CI. This trend is in agreement with the statement that the higher dependence on LD in searching for dominance and epistatic effects compared to additive effects significantly decreases the chance of validating associations in two independent populations for non-additive effects of the markers [48]. Failure to validate many associations could also be related to the genetic differences between breeds, or even populations, and the fact that many QTLs are only segregating in one breed and not in the other [33, 41]. In situations like these, the validation may not be successful in confirming a true positive that exists in one breed but not shared between breeds, even if the power for detecting associations in both populations is high. Detecting marker effects and validating them on very dense genotypes or sequence data may help to overcome these problems.

Quantifying non-additive gene actions requires phenotypes that are measured on genotyped individuals. Daughter yield deviations are performance averages typically over hundreds of daughters. However, by definition they cannot capture the dominance deviation in daughters’ phenotypes, so they are not useful for estimating non-additive effects of genes. The only way to estimate non-additive genetic effects in dairy cattle is through large datasets of dairy cows, as they express almost all of the economically important traits in dairying.

The number of cows with available genotypes in dairy cattle is less than the genotyped bulls but the trend is towards genotyping more cows. Moreover, the availability of genotyped ancestors of cows enables inferring genotype probabilities for cows that can be then used in estimation of non-additive effects [51]. Nonetheless, accurate estimation of non-additive genetic effect requires more data than the data needed for additive effects to maintain the same power in analysis [20, 48] and this is exacerbated by the necessity of having observations in all three genotype classes. We removed a large number of SNPs with minor genotypic frequency <0.01 here when included biased estimates of dominance effects were obtained because of a missing class of SNP genotype. A large number of genotyped cows with a high resolution (dense) genome content or whole genome sequence could solve this problem.

## Conclusions

We identified and validated a small number of SNPs with suggested dominance effects on MY and CI in Australian Holstein and Jersey cows. Given our results, identifying non-additive gene actions using single SNP regression in a GWAS setting will require very large datasets to capture the likely very small individual non-additive genetic effects. Alternative approaches that simultaneously use all genomic information in Bayesian methods or in terms of genomic relationship matrices in a BLUP seem more appropriate. As the number of genotyped animals is steadily increasing in dairy cattle breeding, incorporating whole genome sequence could reduce the problem of high dependency on LD in detection of non-additive genetic effects and is suggested as a future approach.

## Methods

### Data

**Fig. 5:**
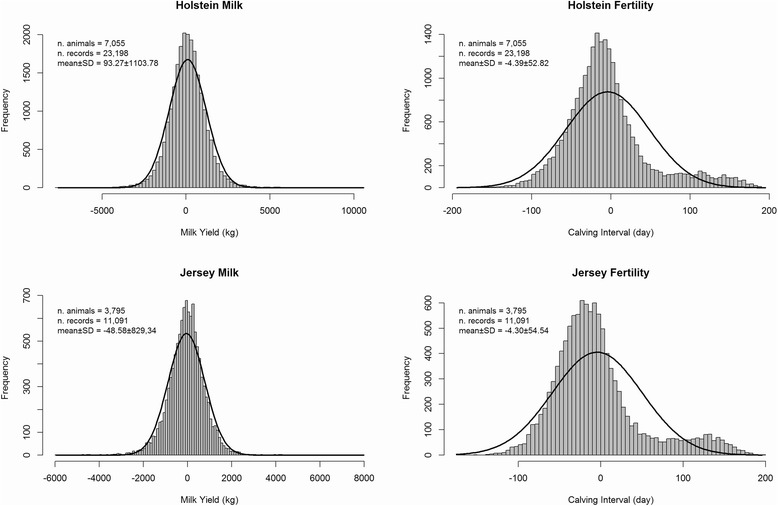
Summary statistics of data. Distribution and summary statistics of Holstein and Jersey datasets for milk yield and calving interval

The original dataset contained 9,159,969 calving interval and 305 d milk yield records of 3,513,757 Australian Holstein and Jersey cows that were in lactation between 1980 and 2011 and recorded by the Australian Dairy Herd Improvement Scheme (ADHIS; Melbourne, Australia). Not all contemporaries in a given herd-year-season were genotyped, and therefore, to accurately remove contemporary group effects from the phenotypes, the records were pre-corrected for the effects of age at calving, herd-year-season, parity and month of calving using the full set of records. The residuals from this model were used as the response variable in GWAS analyses for the genotyped animals. Records that did not have both genotype and phenotype data were discarded, so that the final datasets included 23,198 and 11,091 MY and CI records respectively for 7,055 Holstein and 3,795 Jersey cows. Distribution and descriptive statistics of the Holstein and Jersey populations’ phenotypes are shown in Fig. 5.

The genotyped animals were part of the Australian genomic reference population that were previously selected for typing based on completeness of their phenotypic data and having sires in the national Australian genomic reference population. Animals were genotyped with Illumina BovineSNP50 v2 BeadChip (Illumina, San Diego, CA, USA) and their 50 K SNP data were imputed to the high density (HD) 800 k panel by using another set of 1,785 animals (including Holstein and Jersey key ancestor bulls and heifers) with genotypes from the Illumina BovineHD BeadChip (Illumina, San Diego, CA, USA). The imputation was performed with BEAGLE 3.1 [52]. Quality control checks applied on both genotypic data sets prior to the imputation step were as described in Erbe *et al.* [53]: only SNPs with Gen-Call score > 0.6 and call rate > 90 % were kept; mitochondrial, unmapped and duplicate map position SNPs were removed; and a minimum number of 10 copies for the minor allele was required for each SNP to be included in the data set. This resulted in 45,754 and 632,003 SNPs for the 50 K and 800 K panels, respectively. A further 223,748 SNPs were removed from HD SNP panel owing to a genotype class had a frequency <0.01 in both Holstein or Jersey animals. This was done because the scope of this study focused on both additive and dominance effects of the SNPs and required enough observations in all three classes of SNP genotypes. Finally, the remaining 408,255 SNPs were used for the GWAS. The positions of SNPs on the genome were based on UMD 3.1 genome assembly [54] and the coding of SNP genotypes were 0, 1, and 2 respectively for aa, Aa and AA allele combinations. More details on filtering and imputation of SNPs can be found in Erbe *et al.* [53] and Hayes *et al.* [55].

A total of 408,255 SNPs included in the analyses were distributed over 29 BTA as well as the X chromosome, but the distribution was uneven with an average genome-wide distance of 6.52 Kb between SNPs, and a minimum and maximum average distance of 5.40 Kb and 15.37 Kb on BTA 25 and on X chromosome, respectively.

### Statistical model

A mixed linear model was implemented to test for associations between genetic markers and phenotypic values of both traits (CI and MY) for each breed separately. SNP effects were calculated one at a time using single SNP regression procedure. The model in matrix notation was:

1 $$ \mathbf{y}={1}_{\mathbf{n}}\mu +\mathbf{X}\mathbf{b}+\mathbf{Z}\mathbf{u}+\mathbf{W}\mathbf{p}\mathbf{e}+\mathbf{e} $$

where **y** is a vector of phenotypes (CI or MY), **1**_**n**_ is a vector of ones, μ is the population mean term, **b** is the vector containing relevant additive, dominance or epistatic marker effects as specified below, **u** contains polygenic effects assumed to be distributed as **u** ~ N(0, **A***σ*_*g*_^2^) with **A** being the pedigree based numerator relationship matrix, **pe** is the vector of random permanent environmental effects with **pe** ~ N(0, **I***σ*_*pe*_^2^) and **e** is a vector of random residual deviates supposed to be distributed as **e** ~ N(0, **I***σ*_*e*_^2^). **X** is a design matrix allocating records to markers effects (additive, dominance or epistatic) and **Z** and **W** are incidence matrices for the random effects. *σ*^*2*^_*g*_, *σ*^*2*^_*pe*_ and *σ*^*2*^_*e*_ are polygenic additive, permanent environmental and residual variances, respectively. The pedigree files included 29,042 and 15,977 animals for Holstein and Jersey with corresponding average generations of seven and six.

The original marker covariates (0, 1 or 2) were corrected for allele frequencies [13] to build **X**, so that *x*_*ij*(*a*)_ = {−2*p*, (*q* − *p*) *or* 2*q*} for additive effects of aa, Aa and AA genotypes, respectively, with *p* and *q* being the allele frequency of A and a allele at marker *j* in the population. For dominance effects, aa, Aa and AA genotypes were coded as *x*_*ij*(*d*)_ = {−2*p*^2^, 2*pq* and − 2*q*^2^}. Then, the contents of **Xb** varied with the type of the genetic effect being tested. For additive effects, **Xb** = {*x*_*ij*(*a*)_*a*_*j*_}, where *a*_*j*_ is the corresponding additive effect. For dominance, **Xb** = {*x*_*ij*(*a*)_*a*_*j*_ + *x*_*ij*(*d*)_*d*_*j*_}, where *d*_*j*_ is the dominance effect of marker *j*. In the epistasis model, $\mathbf{X}\mathbf{b}=\left\{{x}_{ij(a)}{a}_j+{x}_{ij{}^{\prime }(a)}{a}_{j{}^{\prime }}+{x}_{ijj{}^{\prime }(e)}{a}_{jj{}^{\prime }}\right\},$ where ${x}_{ij{j}^{\prime }(e)}$ is the qualification for nested interaction effects involving markers *j* and *j’*, ${a}_{j^{\prime }}$ is the corresponding additive effect for the *j’* marker and ${a}_{j{j}^{\prime }}$ is the pairwise additive by additive epistatic marker effect between markers *j* and *j’*. The models were fitted to the data with ASReml v3.0 [56].

### Validation

#### Cross validation

To discriminate between true discoveries and false positive SNP effects, and also to confirm significant SNPs that were consistent between breeds, results from the Holstein and Jersey data sets were compared. The larger Holstein population was assigned to the discovery set and results from Holstein analyses were validated in the Jersey breed in two different ways. First, if a significant SNP was found in the discovery process, we examined whether it was also significant in the validation population. Second, for each significant SNP in the discovery population, we searched for significant SNPs in the validation population within the region 500 kb downstream and upstream of the identified SNP. We call the latter segment validation which accounts for the possible difference in allele frequency of markers in LD with the causal mutation in the different population, that may cause different markers in the same region are tagging the same causal mutation in different populations. The window size was chosen by considering the long range of strong LD between SNPs in the Holstein and Jersey breeds [50, 57].

### False Discovery Rate (FDR)

The false discovery rate was calculated following the approach proposed by Bolormaa *et al.* [58].

2 $$ \%FDR=\frac{P\left(1-\frac{S}{T}\right)}{\left(\frac{S}{T}\right)\left(1-P\right)}\times 100 $$

where *P* is the *P-*value threshold in *F-*test, *S* is the number of significant SNPs according to this threshold and *T* is the total number of tests. This FDR can be used to provide an expectation of the number of true positives at the probability thresholds.

The *P-*value level used to decide on significance of a main or interaction effect was the corresponding Bonferroni correction of the nominal *P-*value of 0.05 as: $\frac{0.05}{Number\  of\  tests},$ so the adjusted *P*-value threshold in the analysis including all SNPs was equal to *P* < 1 × 10^−7^. In the case that no significant SNP effects were identified at this threshold, an arbitrary *P-*value threshold of *P* < 0.0001 in the discovery dataset (*i.e.* Holsteins) and *P* < 0.01 in the validation population (*i.e.* Jerseys) was used. Although this might be arbitrary and some false positives may arise using this approach, it is highly unlikely for significant effects to arise simultaneously in two completely independent populations and our ultimate standard is across breed validation.

For additive and dominance models, all of the markers in the final HD panel were used. To reduce the dimension of SNP combinations tested in the epistatic models, only significant SNPs determined using the *P*-value of the *F-*test of the additive model in the Holstein discovery set were included. Therefore, *n*(*n*-1)/2 pairwise interactions were tested where *n* is the number of SNPs whose main additive effect is significant.

The additive (*σ*_*a*_^2^) or dominance (*σ*_*d*_^2^) variance of each SNP was estimated as follows:

3 $$ {\sigma}_{a_i}^2=2{p}_i{q}_i{a}_i^2 $$

4 $$ {\sigma}_{d_i}^2=4{p}_i^2{q}_i^2{d}_i^2 $$

where *p*_*i*_ and *q*_*i*_ are the frequency of A and a alleles, and *a*_*i*_ and *d*_*i*_ are respectively the estimated additive and dominance effect of the *i*th SNP obtained from model (1) and model (2). For most strongly associated additive or dominance SNP effects, these variances were expressed as a fraction of total phenotypic variance (*σ*_*p*_^2^ = *σ*_*g*_^2^ + *σ*_*pe*_^2^ + *σ*_*e*_^2^) where each variance component was estimated based on model (1) without fitting SNP covariates.

Manhattan plots were created using qqman 0.1.1 [59] in R 3.1.0 (R Development Core, 2014) and the National Center for Biotechnology Information’s databases (www.ncbi.nlm.nih.gov) were used to determine if the significant SNPs were positioned inside known genes. Animal genome QTL database (AnimalQTLdb: http://www.animalgenome.org/cgi-bin/QTLdb/index) was used to compare the results of present study with the reported QTL regions in the literature.

## Additional file

Additional file 1:
**Candidate genes for milk yield.** **Table S1.** Positions of genes associated with individually validated SNPs with additive effects on milk yield.

## Abbreviations

SNP: Single nucleotide polymorphism
BTA: *Bos taurus* autosome
CI: Calving interval
GWAS: Genome-wide association study
LD: Linkage disequilibrium
MY: Milk yield
FDR: False discovery rate
Mbp: Mega base pair
Kbp: Kilo base pair
GC: Granulosa cell
HD: High density
