# Supplementary material for: Validation of markers with non-additive effects on milk yield and fertility in Holstein and Jersey cows
Source: BMC Genet. 2015 Jul 22;16:89. doi: 10.1186/s12863-015-0241-9 (PMC4509610; doi:10.1186/s12863-015-0241-9)
Supplement: Additional file 1: — Candidate genes for milk yield. Table S1. Positions of genes associated with individually validated SNPs with additive effects on milk yield. [file 12863_2015_241_MOESM1_ESM.pdf]

Table S1 Base pair (bp) positions of genes associated with individually validated SNPs with additive effects on milk yield

Gene name	Start (bp)	End (bp)	Description[1]
GPRC5A	97433255	97453094	Bos taurus G protein-coupled receptor, family C, group 5, member A (GPRC5A), mRNA. [Source:RefSeq mRNA;Acc:NM_001034515]
ARHGAP39	1563866	1600378	Rho GTPase activating protein 39 [Source:HGNC Symbol;Acc:HGNC:29351]
PPP1R16A	1628814	1633988	Bos taurus protein phosphatase 1, regulatory subunit 16A (PPP1R16A), mRNA. [Source:RefSeq mRNA;Acc:NM_001193251]
CYHR1	1663923	1677519	Bos taurus cysteine/histidine-rich 1 (CYHR1), transcript variant 1, mRNA. [Source:RefSeq mRNA;Acc:NM_001015532]
TONSL	1681494	1692498	Bos taurus tonsoku-like, DNA repair protein (TONSL), mRNA. [Source:RefSeq mRNA;Acc:NM_001075188]
VPS28	1693641	1698490	Vacuolar protein sorting 28 homolog (S. cerevisiae) [Source:HGNC Symbol;Acc:HGNC:18178]
CPSF1	1728207	1742670	Bos taurus cleavage and polyadenylation specific factor 1, 160kDa (CPSF1), mRNA. [Source:RefSeq mRNA;Acc:NM_174720]
DGAT1	1795351	1804562	Diacylglycerol O-acyltransferase 1 [Source:RefSeq peptide;Acc:NP_777118]
MROH1	1844664	1894424	Maestro heat-like repeat family member 1 [Source:HGNC Symbol;Acc:HGNC:26958]
KIAA1875	1913048	1921667	[Source:HGNC Symbol;Acc:HGNC:26959]
MAF1	1921784	1924818	Bos taurus MAF1 homolog (S. cerevisiae) (MAF1), mRNA. [Source:RefSeq mRNA;Acc:NM_001034424]
GPAA1	1942672	1945910	Bos taurus glycosylphosphatidylinositol anchor attachment protein 1 homolog (yeast) (GPAA1), mRNA. [Source:RefSeq mRNA;Acc:NM_001024529]
LOC101908059	1990570	2006717	uncharacterized
GRINA	2018559	2021709	Bos taurus glutamate receptor, ionotropic, N-methyl D-aspartate-associated protein 1 (glutamate binding) (GRINA), mRNA. [Source:RefSeq mRNA;Acc:NM_001037593]
SCRIB	2178232	2201042	Scribbled planar cell polarity protein [Source:HGNC Symbol;Acc:HGNC:30377]
LOC506831	2201263	2223864	iQ motif and ankyrin repeat domain-containing protein LOC642574-like
FAM83H	2227350	2232020	Family with sequence similarity 83, member H [Source:HGNC Symbol;Acc:HGNC:24797]
MAPK15	2235034	2240765	Mitogen-activated protein kinase 15 [Source:HGNC Symbol;Acc:HGNC:24667]
PYCRL	2301587	2309099	Bos taurus pyrroline-5-carboxylate reductase-like (PYCRL), mRNA. [Source:RefSeq mRNA;Acc:NM_001014906]
GSDMD	2341290	2346302	Bos taurus gasdermin D (GSDMD), mRNA. [Source:RefSeq mRNA;Acc:NM_001046160]
ZC3H3	2354390	2418557	Zinc finger CCCH-type containing 3 [Source:HGNC Symbol;Acc:HGNC:28972]
LOC511669	2511313	2525898	zinc finger and SCAN domain-containing protein 10-like
GLI4	2525957	2532268	GLI family zinc finger 4
LY6H	2542961	2566799	lymphocyte antigen 6 complex, locus H
LY6E	2624758	2628462	Bos taurus lymphocyte antigen 6 complex, locus E (LY6E), mRNA. [Source:RefSeq mRNA;Acc:NM_001046070]
TSNARE1	3054763	3171546	Bos taurus t-SNARE domain containing 1 (TSNARE1), mRNA. [Source:RefSeq mRNA;Acc:NM_001082446]
LOC101905853	3347622	3367438	uncharacterized
SLC45A4	3711837	3739970	Solute carrier family 45, member 4 [Source:HGNC Symbol;Acc:HGNC:29196]
PTK2	3870893	4065010	Bos taurus PTK2 protein tyrosine kinase 2 (PTK2), mRNA. [Source:RefSeq mRNA;Acc:NM_001075250]
EIF2C2	4085146	4168483	Bos taurus eukaryotic translation initiation factor 2C, 2 (EIF2C2), mRNA. [Source:RefSeq mRNA;Acc:NM_205794]
TRAPPC9	4229512	4616552	Trafficking protein particle complex 9 [Source:HGNC Symbol;Acc:HGNC:30832]

[1] Obtained from www.ensemble.org and www.ncbi.nlm.nih.gov
